# Relationship between the appropriateness of antibiotic treatment and clinical outcomes/medical costs of patients with community-acquired acute pyelonephritis: a multicenter prospective cohort study

**DOI:** 10.1186/s12879-022-07097-9

**Published:** 2022-02-01

**Authors:** Choseok Yoon, Se Yoon Park, Bongyoung Kim, Ki Tae Kwon, Seong-yeol Ryu, Seong-Heon Wie, Hyun-uk Jo, Jieun Kim, Kyung-Wook Hong, Hye In Kim, Hyun ah Kim, Mi-Hee Kim, Mi-Hyun Bae, Yong-Hak Sohn, Jieun Kim, Yangsoon Lee, Hyunjoo Pai

**Affiliations:** 1 The Medical Commend of Internal Medicine, 27th Infantry Division Medical Team, Republic of Korea Army, Hwacheon, Korea; 2 Division of Infectious Diseases, Department of Internal Medicine, Soonchunhyang University Seoul Hospital, Soonchunhyang University College of Medicine, Seoul, Korea; 3 Department of Internal Medicine, Hanyang University College of Medicine, 222-1, Wangsimni-ro, Seongdong-gu, Seoul, 04763 Korea; 4 Department of Internal Medicine, School of Medicine, Kyungpook National University, Daegu, Korea; 5 Department of Internal Medicine, Keimyung University Dongsan Medical Center, Daegu, Korea; 6 Division of Infectious Diseases, Department of Internal Medicine, St. Vincent Hospital, College of Medicine, The Catholic University of Korea, Seoul, Korea; 7 Department of Urology, Good Moonhwa Hospital, Busan, Korea; 8 Division of Infectious Diseases, Department of Internal Medicine, Gyeongsang National University Hospital, Gyeongsang National University School of Medicine, Jinju, Korea; 9 Department of Internal Medicine, Daegu Fatima Hospital, Daegu, Korea; 10 Department of Laboratory Medicine, College of Medicine, Hanyang University, Seoul, Korea; 11 Seegene Medical Foundation, Seoul, Korea; 12 Department of Laboratory Medicine, Soonchunhyang University Seoul Hospital, Soonchunhyang University College of Medicine, Seoul, Korea

**Keywords:** Acute pyelonephritis, Urinary tract infection, Antibiotics, Korea

## Abstract

**Background:**

Inappropriate use of antibiotics not only increases antibiotic resistance as collateral damage but also increases clinical failure rates and medical costs. The purpose of this study was to determine the relationship between the appropriateness of antibiotic prescription and outcomes of community-acquired acute pyelonephritis (CA-APN).

**Methods:**

A multicenter prospective cohort study was conducted at eight hospitals in Korea between September 2017 and August 2018. All hospitalized patients aged ≥ 19 years who were diagnosed with CA-APN on admission were recruited. The appropriateness of empirical and definitive antibiotics, as well as the appropriateness of antibiotic treatment duration and route of administration, was evaluated in accordance with the guideline and expert opinions. Clinical outcomes and medical costs were compared between patients who were administered antibiotics ‘appropriately’ and ‘inappropriately.’

**Results:**

A total of 397 and 318 patients were eligible for the analysis of the appropriateness of empirical and definitive antibiotics, respectively. Of them, 10 (2.5%) and 18 (5.7%) were administered ‘inappropriately’ empirical and definitive antibiotics, respectively. Of the 119 patients whose use of both empirical and definitive antibiotics was classified as ‘optimal,’ 57 (47.9%) received antibiotics over a longer duration than that recommended; 67 (56.3%) did not change to oral antibiotics on day 7 of hospitalization, even after stabilization of the clinical symptoms. Patients who were administered empirical antibiotics ‘appropriately’ had shorter hospitalization days (8 vs. 10 days, *P* = 0.001) and lower medical costs (2381.9 vs. 3235.9 USD, *P* = 0.002) than those who were administered them ‘inappropriately.’ Similar findings were observed for patients administered both empirical and definitive antibiotics ‘appropriately’ and those administered either empirical or definitive antibiotics ‘inappropriately’.

**Conclusions:**

Appropriate use of antibiotics leads to better outcomes, including reduced hospitalization duration and medical costs.

**Supplementary Information:**

The online version contains supplementary material available at 10.1186/s12879-022-07097-9.

## Background

Inappropriate use of antibiotics not only increases antibiotic resistance as collateral damage but also increases clinical failure rates and medical costs [1]. Once a pathogen acquires antimicrobial resistance, it erodes the effectiveness of antibiotics, leads to clinical failure, and increases medical costs [2]. To tackle such a vicious cycle, the antimicrobial stewardship program (ASP)—a set of multidisciplinary activities focused on the proper use of antimicrobials—has been established [3]. Despite this, 20–40% of antibiotic prescriptions are considered inappropriate in hospitals [3].

Urinary tract infection (UTI) is one of the most common community-acquired bacterial infections requiring antibiotic treatment [4]. Acute pyelonephritis (APN) is an upper UTI commonly caused by gram-negative Enterobacterales; *Escherichia coli* is the most common causative organism [5]. It usually responds well to antibiotic treatment; however, the infection can sometimes lead to severe complications or death [6]. Therefore, the use of appropriate antibiotics against the causative pathogen is important for achieving favorable treatment results.

Treatment guidelines for UTIs are developed to help practitioners perform an evidence-based antimicrobial treatment [7, 8], and adherence to them is important not only for better clinical outcomes but also for reducing medical costs and the hospitalization period [9, 10]. Despite this, there are cases of inappropriate use of antibiotics for various reasons, such as concerns about antimicrobial resistance and fear of worsening symptoms [11, 12]. In Korea, the prescription of broad-spectrum antibiotics increased significantly for the treatment of APN; in particular, the use of carbapenem increased approximately threefold during 2010‒2014 [13]. However, the appropriateness of antibiotic prescription for APN has not yet been evaluated in Korea. In addition, there are limited data on the effect of the appropriateness of antibiotic prescription on clinical outcomes and medical costs.

The purpose of this study was to evaluate the appropriateness of antibiotic use for the treatment of community-acquired APN (CA-APN) and determine the relationship between the appropriateness of antibiotic treatment and outcomes of patients with CA-APN.

## Methods

### Study setting

This was a prospective observational multicenter cohort study conducted in Korea. Eight hospitals with 580‒915 beds participated in the study, from September 1, 2017, to August 31, 2018. Participating hospitals were located throughout the Korean peninsula, with most being university-affiliated hospitals (seven out of the eight hospitals). The study protocol was approved by the Institutional Review Board (IRB) of Hanyang University Seoul Hospital (IRB number: 2017-07-009) and the IRB of each hospital. Written informed consent was obtained from the patients by the researchers at each hospital. The study was performed in accordance with the Declaration of Helsinki and all methods were performed in accordance with the relevant guidelines and regulations.

### Patient population

All adult patients (aged ≥ 19 years) with CA-APN admitted to participating hospitals were recruited. The inclusion criteria were fever (body temperature ≥ 37.8 ℃) with the fulfillment of at least three of the following criteria: (i) flank pain; (ii) costovertebral angle tenderness; (iii) symptoms of lower UTI, such as dysuria, urgency, frequency, and suprapubic pain; (iv) pyuria (≥ 5‒9 white blood cells [WBCs] per high power field); and (v) leukocytosis (WBC count > 11,600/mm^3^ or polymorphonuclear cells plus bands > 65%) [14, 15]. CA-APN was defined as a case presenting to the emergency department or an outpatient department from the community with signs of APN as previously described. Patients diagnosed with APN > 48 h after admission, those transferred from other hospitals, who had other reasons for fever and pyuria, with insufficient data, or pregnant women were excluded from the study. Furthermore, patients with prolonged hospitalization (≥ 21 days) due to medical problems not associated with APN treatment, or those whose entire treatment progress had not been observed, were also excluded.

### Clinical data

We collected clinical data on demographic features (age and sex), clinical features, clinical outcomes, and medical costs. To assess outcomes, we recorded the length of hospital stay and clinical failure rate. Clinical failure was defined as death or recurrence of APN within 14 days of completing therapy. For the analysis of medical costs, the costs incurred during hospitalization were extracted from the hospital’s financial database. It consisted of costs such as consultation fees, hospitalization expenditures, meals, cost per medication, procedure or operation charges, laboratory examination charges, and radiologic examination charges. Non-reimbursed medical costs were excluded. All costs are presented in USD (1 USD = 1100 KRW).

### Microbiological data

We analyzed results of the urine and blood culture tests performed at the time of admission. The presence of etiologic agents was confirmed when microorganisms at a concentration of ≥ 10^5^ CFU/mL were isolated from urine cultures and/or when urinary pathogens were isolated from blood cultures. Identification of bacterial species and their susceptibility to antibiotics were determined using a semi-automated system (VITEK, bioMérieux, Hazelwood, MO, USA or Microscan, Dade Behring, West Sacramento, CA, USA) in each hospital. The breakpoints of each compound were defined with reference to the Clinical and Laboratory Standards Institute [16], and the breakpoints of R (resistance) or I (intermediate) were considered to indicate resistance. If the causative organism was resistant to ciprofloxacin and/or levofloxacin, we defined it as resistant to fluoroquinolones.

### Definition of the appropriateness of antibiotic use

The appropriateness of the use of empirical and definitive antibiotics was classified as ‘optimal,’ ‘suboptimal,’ and ‘inappropriate’; ‘optimal’ was regarded as ‘appropriate’ antibiotics use, whereas ‘suboptimal’ and ‘inappropriate’ were regarded as ‘inappropriate’ antibiotic use.

The standard for the classification of empirical antibiotics was defined based on the ‘Clinical Practice Guideline for the Antibiotic Treatment of Community-Acquired Urinary Tract Infections’ which is the national clinical practice guideline in Korea [8]. ‘For the definition of optimal empirical antibiotic treatment, the empirical treatment may have consisted of one of the following antibiotics: second to fourth generation cephalosporins, aminoglycosides, β-lactamase/β-lactamase inhibitors, or fluoroquinolones, regardless of the severity of APN or history of patients. As for carbapenem use, we considered it to be an ‘optimal’ empirical antibiotic use when carbapenem was administered to patients with signs of septic shock (systolic blood pressure < 90 mmHg or mental change), a history of antibiotic treatment, or a history of hospitalization within 1 year [17]. The use of carbapenem in the other cases was regarded as ‘suboptimal’ empirical antibiotic use. The antibiotics not included in the ‘optimal’ or ‘suboptimal’ empirical antibiotic use groups for CA-APN were defined as ‘inappropriate.’

The standard for the classification of definitive antibiotics was defined according to the results of the in vitro susceptibility test for causative organisms. Cases without the result of the causative organism or those with multidrug-resistant *Pseudomonas aeruginosa*—not susceptible to all antibiotics—as the causative pathogen, were excluded. We compared the antibiotics used on the 5th day after urine and/or blood cultures were prepared with the results of the antimicrobial susceptibility test. The ranks of the spectrum of β-lactam and anti-staphylococcal antibiotics are shown in Table 1 [18]. Rank 1 was defined as the narrowest antibiotic, and rank 3 was defined as the broadest antibiotic.

**Table 1:** The rank of the spectrum

	β-lactam antibiotics	Anti-staphylococcal antibiotics
Rank 1	3rd generation cephalosporin (without anti-pseudomonal activity), Ureido/carboxy-penicillin	Nafcillin/Oxacillin, 1st generation cephalosporin
Rank 2	Piperacillin/tazobactam, Ticarcillin/clavulanate, 4th generation cephalosporin, 3rd generation cephalosporin (with anti-pseudmonal activity)	Vancomycin, Teicoplanin, Linezolid
Rank 3	Ertapenem, Imipenem, Meropenem, Doripenem	–

Rank 1 was defined as the narrowest antibiotics and Rank 3 was defined as the broadest one

‘Optimal’ definitive antibiotic use was considered when the causative organisms were susceptible to the antibiotic used on the 5th day. As for the β-lactam and anti-staphylococcal antibiotics, we considered them as ‘optimal’ definitive antibiotics when the causative organism was not ‘susceptible’ to narrower-spectrum antibiotics and ‘suboptimal’ when the causative organism was ‘susceptible’ to narrower-spectrum antibiotics. If the causative organism was not susceptible to the antibiotic that was used on the 5th day, we defined it as an ‘inappropriate’ definitive antibiotic.

Combining the appropriateness of the empirical and definitive antibiotic use, ‘optimal’ antibiotic use was considered when both empirical and definitive antibiotic use was ‘optimal;’ ‘inappropriate’ was considered when at least one of the empirical or definitive antibiotics was ‘inappropriate;’ and ‘suboptimal’ included the remaining cases.

To evaluate the appropriateness of the route of administration, we reviewed the clinical status of patients with a causative organism susceptible to antibiotics capable of oral route administration such as fluoroquinolone, third generation cephalosporin, and trimethoprim/sulfamethoxazole. The criteria for eligibility to use oral antibiotics were as follows: (i) normal WBC count (4500–11,000/µL) and (ii) normal body temperature (36.1‒37.2 °C) on day 7 of hospitalization [19]. ‘Appropriate’ oral antibiotic use was considered when patients who met the abovementioned criteria used oral antibiotics on the 7th day of hospitalization, and ‘inappropriate’ oral antibiotic use was considered when patients who met the criteria did not use oral antibiotics on the 7th day of hospitalization.

Regarding the duration of antibiotic use, ‘prolonged’ antibiotic duration was defined as > 14 days for simple APN cases; > 14 days from the procedure for complicated APN cases; and > 28 days for renal abscess, cyst infection, and emphysematous pyelonephritis cases [7].

### Statistical analyses

Clinical outcomes and medical costs were compared between patients who were administered antibiotics ‘appropriately’ and ‘inappropriately.’ All statistical analyses were conducted using SPSS version 21 for Windows (IBM Corp., Armonk, NY, USA). Categorical variables were analyzed using Fisher’s exact test, and continuous variables were analyzed using the Mann–Whitney test. We also performed a propensity-score matching analysis with a matching weight of 1:1 or 1:2 to reduce the effects of confounding factors. Age, sex, Charlson comorbidity index, and Pitt bacteremia score were included in the model, and non-matched cases were discarded for the subanalysis. Statistical significance was set at a two-tailed *P*-value < 0.05.

## Results

### Patient population and baseline characteristics

A total of 419 patients with CA-APN were enrolled during the study period. Among them, 22 were excluded for the following reasons: 3 were pregnant, 6 were hospitalized for ≥ 21 days owing to medical problems not associated with APN treatment, and 13 were those whose entire treatment progress was not observed. Finally, 397 patients were included in the analysis.

Table 2 presents the baseline patient characteristics. The mean age was 57.63 ± 18.78 years, and 92.9% were women. The mean Charlson comorbidity index was 0.91 ± 1.33, and 30.5% were diabetic. The mean Pitt’s score was 0.65 ± 0.87, and 44.8% had bacteremia. Of the 322 cases in which the causative organisms were identified, *E. coli* was the most common causative organism (88.8%), followed by *Klebsiella* spp. (4.3%) and *Enterobacter* spp. (1.2%). As for antimicrobial therapy, third or fourth generation cephalosporins (65.2%) were mostly chosen as the initial antimicrobial regimen, followed by fluoroquinolone (18.6%), beta-lactam/beta-lactamase inhibitor (9.1%), and carbapenem (5.3%). Of the 318 patients for whom the antimicrobial susceptibility result of the causative organism was available, 23.6% received initial empirical antibiotics that were discordant to the antimicrobial susceptibility of the causative pathogens. The median duration of antibiotic therapy was 9 days, and 10.6% received surgical procedures including nephrostomy as treatment for APN.

**Table 2:** Baseline characteristics of patients with community-acquired acute pyelonephritis

	Total (n = 397)
Demographic data	
Age (years), mean ± SD	57.63 ± 18.78
Female sex (%)	369 (92.9)
Underlying co-morbidities	
Charlson’s comorbidity index, mean ± SD	0.91 ± 1.33
Diabetes mellitus (%)	121 (30.5)
Cerebrovascular accident (%)	33 (8.3)
Malignancy (%)	27 (6.8)
Renal disease (%)	27 (6.8)
Dementia (%)	18 (4.5)
Bedridden state (%)	9 (2.3)
Any structural problems on urinary tract (%)	18 (4.5)
Past history	
History of admission during 1 year prior to inclusion (%)	87 (21.9)
History of antibiotic usage during 1 year prior to inclusion (%)	110 (27.7)
History of urinary tract infection during 1 year prior to inclusion (%)	38 (9.6)
Clinical characteristics	
Pitt’s score, mean ± SD	0.65 ± 0.87
Hematuria (%)	207 (52.1)
Azotemia (%)	110 (27.7)
Bacteremia (%)	176/393 (44.8)
Causative organisms (%)	
*Escherichia coli*	286/322 (88.8)
*Klebsiella* spp.	14/322 (4.3)
*Enterobacter* spp.	4/322 (1.2)
*Proteus* spp.	3/322 (0.9)
*Enterococcus* spp.	3/322 (0.9)
*Pseudomonas* spp.	3/322 (0.9)
*Citrobacter* spp.	2/322 (0.6)
Others	7/322 (2.2)
Antimicrobial therapy	
Initial antimicrobial regimen (%)	
3rd generation or 4th generation cephalosporin	259 (65.2)
Fluoroquinolone	74 (18.6)
Beta-lactam/beta-lactamase inhibitor	36 (9.1)
Carbapenem	21 (5.3)
Discordant to the antimicrobial susceptibility of causative organism	75/318 (23.6)
Per oral antibiotics	0 (0)
Duration of total antibiotics, days, median (IQR)	9 (7–12)
Surgical procedures as a treatment of acute pyelonephritis (%)	42 (10.6)

SD, standard deviation; IQR, interquartile rang

### Clinical outcomes and medical costs according to the appropriateness of antibiotic use

A total of 397 and 318 patients were eligible for the analysis of the appropriateness of empirical and definitive antibiotics, respectively. Of them, 14 (3.5%) and 69 (21.7%) were classified as patients who were administered ‘inappropriately’ empirical and definitive antibiotics, respectively; 81 (25.5%) were administered either empirical or definitive antibiotics inappropriately.

**Fig. 1:**
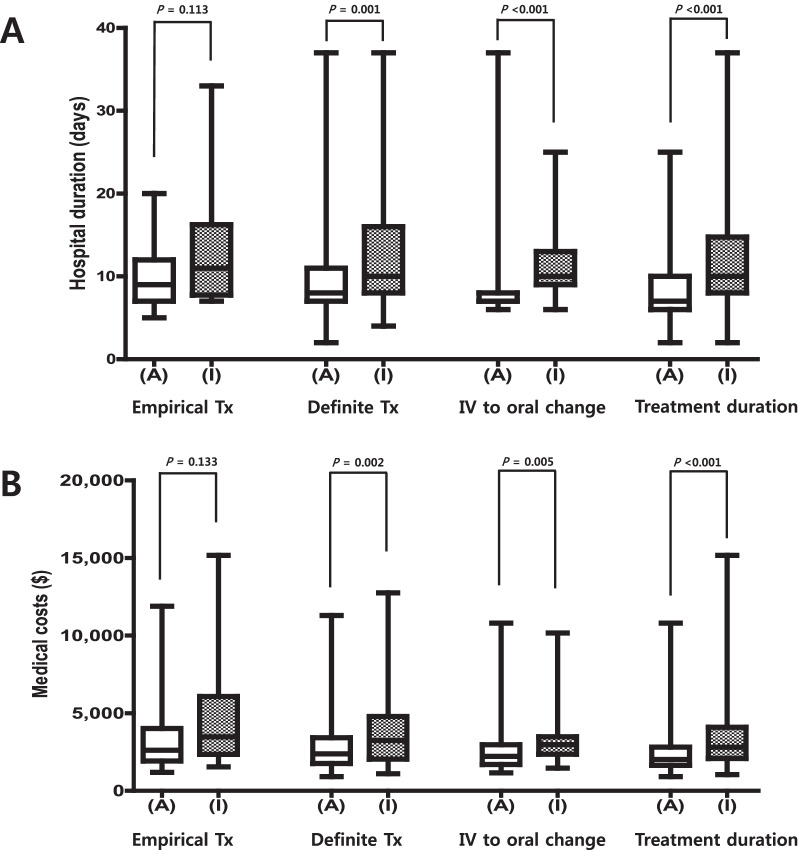
Clinical outcomes and medical costs according to the appropriateness of antibiotic use. **A** Hospital duration (days). **B** Medical costs (USD). Tx, treatment; IV, intravenous

After propensity-score matching, it did not show significance between patients administered empirical antibiotics ‘appropriately’ and ‘inappropriately’. In comparison, patients who were administered definite antibiotics ‘appropriately’ had shorter hospitalization days (8 vs. 10 days, *P* = 0.001), and lower medical costs (2,381.9 vs. 3,235.9 USD, *P* = 0.002) than those who were administered them ‘inappropriately.’ Similarly, Patients administered both empirical and definitive antibiotics ‘appropriately’ had lower medical costs (2,373.6 vs. 3,190.8 USD, *P* < 0.001) than those administered either empirical or definitive antibiotics ‘inappropriately’ (Table 3A and Fig. 1). According to the analyses before propensity-score matching, the overall results were similar to those of after propensity-score matching, but patients who were administered empirical antibiotics ‘appropriately’ had lower mortality rates, shorter hospitalization days, and lower medical costs than those who were administered them ‘inappropriately’ (Additional file 1: Table S1A).

**Table 3:** Clinical outcomes and medical costs of community-acquired acute pyelonephritis according to the appropriateness of antibiotic use: after propensity-score matching

A. Empirical and definitive therapy									
	Empirical therapy			Definitive therapy			Empirical & definitive therapy		
	Appropriate (n = 28)	Inappropriate^a^ (n = 14)	*P*	Appropriate (n = 138)	Inappropriate^a^ (n = 69)	*P*	Appropriate (n = 162)	Inappropriate^a^ (n = 81)	*P*
Clinical failure (%)	0 (0)	1 (7.1)	0.333	0 (0)	2 (2.9)	0.110	0 (0)	2 (2.5)	0.110
Mortality	0 (0)	1 (7.1)	0.333	0 (0)	1 (1.4)	0.333	0 (0)	1 (1.2)	0.333
Recurrence	0 (0)	0 (0)	NA	0 (0)	1 (1.4)	0.333	0 (0)	1 (1.2)	0.333
Hospitalization days, median (IQR)	9 (7–12)	11 (7.75–16.25)	0.113	8 (7–11)	10 (8–16)	0.001	8 (6.75–11)	8 (11–16)	< 0.001
Medical costs, USD, median (IQR)	2619.3 (1916.8–4006.1)	3477.2 (2341.0–6072.9)	0.133	2381.9 (1758.8–3416.8)	3235.9 (2038.2–4785.6)	0.002	2373.6 (1726.3–3444.7)	3190.8 (2101.2–4837.1)	< 0.001

B. Intravenous to oral antibiotic switch and duration of antibiotic therapy						
	Intravenous to oral antibiotic switch			Duration of antibiotic therapy		
	Appropriate (n = 43)	Inappropriate^a^ (n = 43)	*P*	Appropriate (n = 188)	Inappropriate^a^ (n = 188)	*P*
Clinical failure (%)	0 (0)	2 (4.7)	0.494	3 (1.6)	3 (1.6)	1.000
Mortality	NA	NA	NA	1 (0.5)	0 (0)	1.000
Recurrence	0 (0)	2 (4.7)	0.494	2 (1.1)	3 (1.6)	1.000
Hospitalization days, median (IQR)	7 (7–8)	10 (9–13)	< 0.001	7 (6–10)	10 (8–14.75)	< 0.001
Medical costs, USD, median (IQR)	2222.3 (1693.2–2960.8)	3021.7 (2365.5– 3513.5)	0.005	2016.9 (1632.8–2814.1)	2804.3 (2074.7–4092.4)	< 0.001

IQR, interquartile range

^a^It includes ‘suboptimal’ and ‘inappropriate’

A total of 130 patients were eligible for the analysis of the appropriateness of intravenous to oral antibiotic switching, and 87 (66.9%) were classified as patients who switched from intravenous antibiotics to oral antibiotics ‘inappropriately’. After propensity-score matching, the ‘appropriate’ group showed significantly shorter hospitalization days (7 vs. 10 days, *P* < 0.001) and lower medical costs (2,222.3 vs. 3,021.7 USD, *P* = 0.005) than the ‘inappropriate’ group. As for the appropriateness of duration of antibiotic therapy, a total of 397 patients were eligible for the analysis, and 188 (47.4%) were considered as patients who were administered antibiotics for an ‘inappropriate’ duration. After propensity-score matching, the ‘appropriate’ group showed significantly shorter hospitalization days (7 vs. 10 days, *P* < 0.001) and lower medical costs (2016.9 vs. 2804.3 USD, *P* < 0.001) than the ‘inappropriate’ group (Table 3B and Fig. 1). According to the analyses before propensity-score matching, the overall results were similar to those of after propensity-score matching (Additional file 1: Table S1B).

### Appropriateness of antibiotic use among patients with CA-APN

**Fig. 2:**
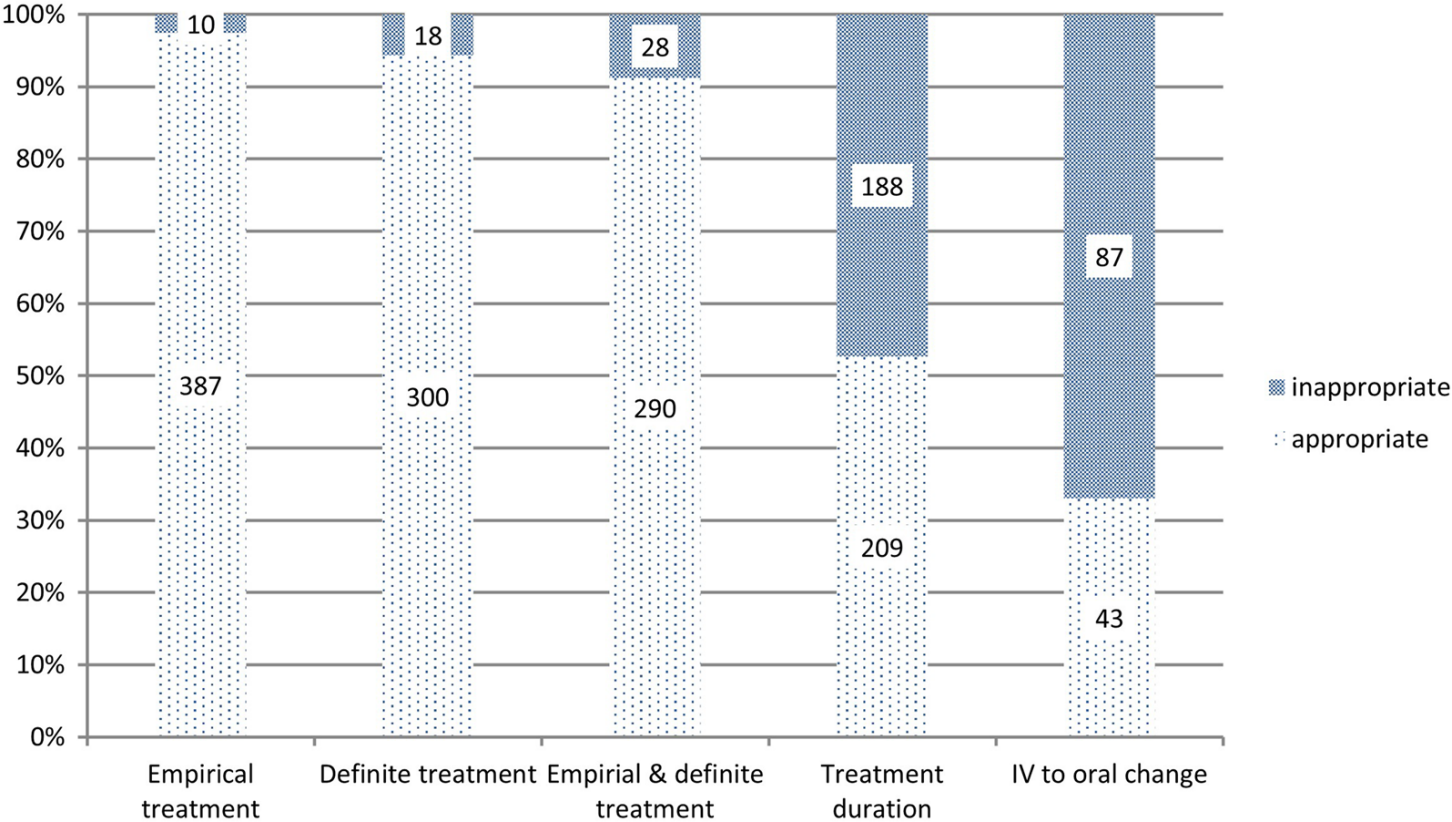
Appropriateness of antibiotic use among patients with community-acquired acute pyelonephritis. IV, intravenous

Table 4 and Fig. 2 show the appropriateness of antibiotic use. Of the 119 patients whose use of both empirical and definitive antibiotics was ‘optimal,’ 57 (47.9%) received antibiotics longer than that recommended; 67 (56.3%) did not change to oral antibiotics on day 7 of hospitalization even after stabilization of the clinical symptoms.

**Table 4:** Appropriateness of antibiotic use among patients with community-acquired acute pyelonephritis

	Optimal			Suboptimal			Inappropriate		
	Total (n = 119)	Appropriate duration (n = 62)	Prolonged antibiotic use (n = 57)	Total (n = 23)	Appropriate duration (n = 6)	Prolonged antibiotic use (n = 17)	Total (n = 10)	Appropriate duration (n = 4)	Prolonged antibiotic use (n = 6)
Patients who met intravenous to oral antibiotic switch criteria on hospital day 7	107 (89.9)	56 (90.3)	51 (89.5)	16 (69.6)	3 (50.0)	13 (76.5)	7 (70.0)	3 (75.0)	4 (66.7)
Change to oral antibiotics	40 (33.6)	25 (40.3)	15 (26.3)	2 (8.7)	0 (0)	2 (11.8)	1 (10.0)	1 (25.0)	0 (0)
Maintenance of parenteral antibiotics	67 (56.3)	31 (50.0)	36 (63.2)	14 (60.9)	3 (50.0)	11 (64.7)	6 (60.0)	2 (50.0)	4 (66.7)
Patients who did not meet intravenous to oral antibiotic switch criteria on hospital day 7	12 (10.1)	6 (9.7)	6 (10.5)	7 (30.4)	3 (50.0)	4 (23.5)	3 (30.0)	1 (25.0)	2 (33.3)

## Discussion

To the best of our knowledge, this is the first study on the relationship between the appropriateness of antibiotic treatment and clinical outcomes/medical costs of patients with infectious diseases in Korea. One of the most significant findings of this study is that inappropriate antibiotic prescription for patients with CA-APN was significantly associated with longer hospitalization duration and higher medical costs. Furthermore, a large proportion of patients, even those administered both empirical and definitive antibiotics appropriately, did not adhere to the recommended treatment duration and did not change to oral antibiotics appropriately.

Several studies have been conducted on the appropriateness of antibiotic prescriptions in patients with UTIs, and the rate of inappropriate antibiotic prescription varies according to the study settings and types of antibiotics evaluated. Regarding the type of empirical antibiotics, a study in the Netherlands found that approximately 30% of empirical antibiotic prescriptions for complicated UTIs did not follow national guidelines [9]. Another study performed in the US revealed that approximately half of the antibiotic prescriptions for uncomplicated UTIs were not first-line agents recommended in the guidelines [11]. In comparison, we found that only 3.5% of empirical antibiotics did not adhere to the national clinical practice guidelines in Korea. The main reason for the low rate of inappropriateness in empirical antibiotics might be that the standards of the Korean guidelines are relatively generous; there is no distinction between first-line and second-line agents [8]. Several studies have reported that inappropriate prescriptions of empirical antibiotics are closely associated with prolonged hospitalization duration when compared with appropriate prescriptions [9, 20]. However, the effect of inappropriate empirical antibiotic prescriptions on mortality remains controversial. Though several studies have shown that inadequate empirical antibiotics for *E. coli* bacteremia increase the risk of mortality, bacteremia caused by UTI is associated with less risk of mortality [21]. According to a Korean study, though discordant empirical antibiotic treatment for UTI leads to worse early clinical response and longer length of hospital stay than concordant treatment, it did not affect overall mortality [22]. Similarly, in our previous study, there was no difference in clinical response, according to the susceptibility to levofloxacin, in patients with UTI caused by *E. coli* (levofloxacin minimal inhibitory concentration < 16 mg/L) and receiving fluoroquinolone as empirical therapy [23]. A possible explanation for this phenomenon is the high urinary concentration of antibiotics, which leads to prolonged bactericidal effects [24].

Interestingly, even though the type of antibiotics was ‘optimal,’ approximately half of the UTI patients were administered antibiotics for a prolonged duration. Similar findings have been reported in other countries. In the US, > 75% of patients with uncomplicated UTIs did not adhere to the recommended antibiotic duration [11]. A retrospective study in the US examined 61 patients with UTIs at an internal medicine clinic and reported that 47% of patients were prescribed an inappropriate antibiotic duration [25]. Another study in the US dealt with patients with uncomplicated UTIs in two private family medicine clinics; > 70% of prescription durations of fluoroquinolone, trimethoprim/sulfamethoxazole, and nitrofurantoin were longer than the guideline recommendations [26]. One possible reason may be that the optimal duration for elderly patients with UTI is not well defined [27]. Another possible reason is that physicians may not pay attention to the evidence supporting shorter antibiotic treatment duration for UTI than for other infectious diseases [28]. In fact, many clinicians may not be familiar with the national clinical practice guidelines because of the difficulty in keeping up with updated recommendations for several different diseases [29]. Implementation of the institution-specific protocol might be helpful in improving each clinician’s awareness of the recommended treatment [30].

In addition to the inappropriate antibiotic duration, not changing to oral antibiotics even after stabilization of the clinical symptoms is another problem. Not only did patient factors such as initial poor condition or delayed response to antibiotics but also clinician factors such as misconception or unawareness of the guidelines delayed the oral switch [31]. Because proper parenteral to oral antibiotic conversion can reduce the workload of nurses, hospitalization days, and medical costs, implementing hospital-wide ASP intervention about the early switch from parenteral to oral antibiotics should be emphasized [32].

There are some potential limitations of our study. First, the standards of ‘inappropriate’ antibiotic use in the present study were relatively generous. For instance, we considered all antimicrobials mentioned in the Korean guideline as ‘appropriate’ regardless of patients’ medical history or clinical status; antimicrobials to which the causative pathogen showed susceptibility, regardless of the antimicrobial spectrum, were considered as ‘appropriate.’ This might be associated with the fact that the current study showed only a small proportion of patients who were administered antibiotics inappropriately. In fact, defining the rank of the spectrum of each antibiotic is a controversial matter and is difficult to classify strictly [18]. In addition, the evaluation timing of appropriateness of route of administration seems a bit late. Because women only require 7 days of treatment, the proportion of inappropriate routes of administration might have been underestimated. Second, some standards of appropriateness in this study may be somewhat controversial. For instance, we did not consider the patients with known ESBL carriage as those who need to use carbapenem as empirical antibiotics, and the empirical use of carbapenem for them was considered as 'suboptimal'. Furthermore, because of the lack of clear standards for ‘inappropriate’ antimicrobial duration or route of administration, we defined them through discussion with researchers. Third, the criteria for APN could be somewhat controversial. According to the criteria used in the present study regarded patients with negative culture results as APN patients. To minimize the possibility of misdiagnosis, patients who had other reasons for fever and pyuria were excluded based on the judgment of experts. Given that there is a possibility of not obtaining the proper culture result for several reasons other than misdiagnosis, our criteria for APN seems to be acceptable. Finally, using Korean data alone is insufficient to shed light on this global problem on the relationship between inappropriate antimicrobial use and unfavorable patient outcomes. Owing to differences in healthcare systems, especially medical costs in each country, our results cannot be generalized to other countries.

## Conclusions

Appropriate use of antibiotics leads to better outcomes, including reduced hospitalization duration and medical costs. ASP should be emphasized to promote appropriate antibiotic prescription in the treatment of UTI.

## Supplementary Information

**Additional file 1: Table S1.** Clinical outcomes and medical costs of community-acquired acute pyelonephritis according to the appropriateness of antibiotic use: before propensity-score matching.

## Data Availability

The datasets used and/or analyzed during the current study are available from the corresponding author on reasonable request.

## Abbreviations

ASP: Antimicrobial stewardship program
UTI: Urinary tract infection
APN: Acute pyelonephritis
CA-APN: Community-acquired acute pyelonephritis
IRB: Institutional Review Board
WBC: White blood cell
