# Supplementary material for: Relationship between the appropriateness of antibiotic treatment and clinical outcomes/medical costs of patients with community-acquired acute pyelonephritis: a multicenter prospective cohort study
Source: BMC Infect Dis. 2022 Feb 1;22:112. doi: 10.1186/s12879-022-07097-9 (PMC8805410; doi:10.1186/s12879-022-07097-9)
Supplement: Supplementary file 1 — Additional file 1: Table S1. Clinical outcomes and medical costs of community-acquired acute pyelonephritis according to the appropriateness of antibiotic use: before propensity-score matching. [file 12879_2022_7097_MOESM1_ESM.docx]

Table S1. Clinical outcomes and medical costs of community-acquired acute pyelonephritis according to the appropriateness of antibiotic use: before propensity-score matching

A. Empirical and Definitive therapy

|  | Empirical therapy | | |  | Definitive therapy | | |  | Empirical & definitive therapy | | |
| --- | --- | --- | --- | --- | --- | --- | --- | --- | --- | --- | --- |
|  | Appropriate  (n = 383) | Inappropriate^a^  (n = 14) | *P* |  | Appropriate  (n = 249) | Inappropriate^a^  (n = 69) | *P* |  | Appropriate  (n = 237) | Inappropriate^a^  (n = 81) | *P* |
| Clinical failure (%) | 5 (1.3) | 1 (7.1) | 0.195 |  | 1 (0.4) | 2 (2.9) | 0.120 |  | 1 (0.4) | 2 (2.5) | 0.161 |
| Mortality | 0 (0) | 1 (7.1) | 0.035 |  | 0 (0) | 1 (1.4) | 0.217 |  | 0 (0) | 1 (1.2) | 0.255 |
| Recurrence | 5 (1.3) | 0 (0) | 0.835 |  | 1 (0.4) | 1 (1.4) | 0.387 |  | 1 (0.4) | 1 (1.2) | 0.445 |
| Hospitalization days, median (IQR) | 9 (7-12) | 11 (7.75-16.25) | 0.018 |  | 8 (7-11) | 10 (8-16) | 0.001 |  | 8 (7-11) | 11 (8-16) | <0.001 |
| Medical costs, USD, median (IQR) | 2,332.7  (1,718.3-3,459.4) | 3,477.2  (2,341.0-6,072.9) | 0.020 |  | 2,371.1  (1,747.2-3,394.0) | 3,235.9  (2,038.1-4,785.6) | 0.001 |  | 2,342.7  (1,723.6-3,385.4) | 3,190.8  (2,101.2-4,837.1) | <0.001 |

B. Intravenous to oral antibiotic switch and duration of antibiotic therapy

|  | intravenous to oral antibiotic switch | | |  | Duration of antibiotic therapy | | |
| --- | --- | --- | --- | --- | --- | --- | --- |
|  | Appropriate  (n = 43) | Inappropriate^a^  (n = 87) | *P* |  | Appropriate  (n = 209) | Inappropriate^a^  (n = 188) | *P* |
| Clinical failure (%) | 0 (0) | 2 (2.3) | 1.000 |  | 3 (1.4) | 3 (1.6) | 1.000 |
| Mortality | NA | NA | NA |  | 1 (0.5) | 0 (0) | 1.000 |
| Recurrence | 0 (0) | 2 (2.3) | 1.000 |  | 2 (1.0) | 3 (1.6) | 0.671 |
| Hospitalization days, median (IQR) | 7 (7-8) | 10 (9-13) | <0.001 |  | 7 (6-10) | 10 (8-14.75) | <0.001 |
| Medical costs, USD, median (IQR) | 2,222.3  (1,693.2-2,960.8) | 2,931.9  (2,308.3- 3,715.5) | 0.002 |  | 2,095.7  (1,646.3-3,016.3) | 2,804.3  (2,074.7-4,092.4) | <0.001 |

Abbreviations: IQR, interquartile range

^a^ It includes ‘suboptimal’ and ‘inappropriate’
